# Estimating the number of cases of podoconiosis in Ethiopia using geostatistical methods

**DOI:** 10.12688/wellcomeopenres.12483.2

**Published:** 2017-12-15

**Authors:** Kebede Deribe, Jorge Cano, Emanuele Giorgi, David M. Pigott, Nick Golding, Rachel L. Pullan, Abdisalan M. Noor, Elizabeth A. Cromwell, Aaron Osgood‐Zimmerman, Fikre Enquselassie, Asrat Hailu, Christopher J. L. Murray, Melanie J. Newport, Simon J. Brooker, Simon I. Hay, Gail Davey

**Affiliations:** 1 School of Public Health, Addis Ababa University, Addis Ababa, Ethiopia; 2 Wellcome Trust Brighton and Sussex Centre for Global Health Research, Brighton and Sussex Medical School, Brighton, UK; 3 London School of Hygiene & Tropical Medicine, London, UK; 4 Lancaster Medical School, Faculty of Health and Medicine, Lancaster University, Lancaster, UK; 5 Institute for Health Metrics and Evaluation, University of Washington, Seattle, WA, USA; 6 Spatial Ecology and Epidemiology Group, Wellcome Trust Centre for Human Genetics, University of Oxford, Oxford, UK; 7 School of BioSciences, University of Melbourne, Parkville, VIC, Australia; 8 Centre for Tropical Medicine and Global Health, Nuffield Department of Clinical Medicine, University of Oxford, Oxford, UK; 9 Kenya Medical Research Institute-Wellcome Trust Collaborative Programme, Nairobi, Kenya; 10 Department of Microbiology, Immunology and Parasitology, Faculty of Medicine, Addis Ababa University, Addis Ababa, Ethiopia; 11 Bill and Melinda Gates Foundation, Seattle, WA, USA; 12 Big Data Institute, Li Ka Shing Centre for Health Information and Discovery, University of Oxford, Oxford, UK

**Keywords:** Ethiopia, podoconiosis, elephantiasis, lymphoedema, neglected tropical disease, mossy foot

## Abstract

**Background**: In 2011, the World Health Organization recognized podoconiosis as one of the neglected tropical diseases. Nonetheless, the  magnitude of podoconiosis and the geographical distribution of the disease is poorly understood. Based on a nationwide mapping survey and geostatistical modelling, we predict the prevalence of podoconiosis and estimate the number of cases across Ethiopia.

**Methods**: We used nationwide data collected in Ethiopia between 2008 and 2013. Data were available for 141,238 individuals from 1,442 communities in 775 districts from all nine regional states and two city administrations. We developed a geostatistical model of podoconiosis prevalence among adults (individuals aged 15 years or above), by combining environmental factors. The number of people with podoconiosis was then estimated using a gridded map of adult population density for 2015.

**Results**: Podoconiosis is endemic in 345 districts in Ethiopia: 144 in Oromia, 128 in Southern Nations, Nationalities and People’s [SNNP], 64 in Amhara, 4 in Benishangul Gumuz, 4 in Tigray and 1 in Somali Regional State. Nationally, our estimates suggest that 1,537,963 adults (95% confidence intervals, 290,923-4,577,031 adults) were living with podoconiosis in 2015. Three regions (SNNP, Oromia and Amhara) contributed 99% of the cases. The highest proportion of individuals with podoconiosis resided in the SNNP (39%), while 32% and 29% of people with podoconiosis resided in Oromia and Amhara Regional States, respectively. Tigray and Benishangul Gumuz Regional States bore lower burdens, and in the remaining regions, podoconiosis was almost non-existent.

**Conclusions**: The estimates of podoconiosis cases presented here based upon the combination of currently available epidemiological data and a robust modelling approach clearly show that podoconiosis is highly endemic in Ethiopia. Given the presence of low cost prevention, and morbidity management and disability prevention services, it is our collective responsibility to scale-up interventions rapidly.

## Introduction

Podoconiosis is an endemic, non-filarial lymphoedema of the lower limb. It affects genetically susceptible individuals who often go barefoot ( Price, 1990). It causes bilateral, often below knee lymphoedema of the lower limb. Based on existing evidence, the most accepted cause of podoconiosis is that of mineral particle-induced inflammation on a background of genetic susceptibility ( Price, 1990). Specific control methods include the use of footwear, regular foot hygiene and floor coverings. For those with the diseases management of the lymphoedema-related morbidity is recommended which includes foot hygiene, foot care, wound care, compression, exercises, elevation of the legs and treatment of acute infections ( Deribe *et al.*, 2015a).

Estimating the number of people with podoconiosis according to geographical location is important for programme planners and health care providers, who plan, monitor and evaluate control and elimination efforts. Despite a growing interest in understanding the number of people with podoconiosis, the figures available are largely estimates ( Davey *et al.*, 2012). Estimation of the number of people with podoconiosis has relied upon expert opinion and a few market-based surveys ( Oomen, 1969; Price, 1974). More robust estimates and a better understanding of how podoconiosis-affected individuals may be spatially distributed are vital ( Deribe *et al.*, 2013; Deribe *et al.*, 2015b). Data on the geographical distribution of podoconiosis are also important to identify populations disproportionately affected by the disease, which need priority intervention.

In our previous analysis, we identified the individual and environmental risk factors of podoconiosis in Ethiopia ( Deribe *et al.*, 2015a; Deribe *et al.*, 2015b), delineated the environmental limits, and estimated the population at risk ( Deribe *et al.*, 2015b). However, the number of people with podoconiosis and its geographical variation remains to be determined. Previous complete censuses of cases have estimated the number of podoconiosis cases in localized areas ( Alemu *et al.*, 2011; Desta *et al.*, 2003; Geshere Oli *et al.*, 2012; Molla *et al.*, 2012; Tekola Ayele *et al.*, 2013). Here we present the results of a modelling exercise intended to analyse the spatial distribution of podoconiosis prevalence across Ethiopia and produce robust estimates of number of cases of podoconiosis for the year 2015. 

## Methods

### Data sources

***Podoconiosis.*** Ethiopia is located in the Horn of Africa and is the second most populated country in sub-Saharan Africa. The total population was estimated at 94.4 million in 2017, of which over 75 million (79.7%) live in rural areas ( Federal Democratic Republic of Ethiopia Ministry of Health, 2016). The data used in this study are derived from nationwide mapping of lymphatic filariasis (LF) and podoconiosis conducted between 2008 and 2013 ( Deribe *et al.*, 2015a; Deribe *et al.*, 2015b). Detailed information about the surveys has been provided elsewhere ( Deribe *et al.*, 2015a; Deribe *et al.*, 2015b). Briefly, the study was conducted in all nine Regional States and two City Administrations (Addis Ababa and Dire Dawa). In each district ( *woreda*), two communities were surveyed purposively following WHO guidelines for LF mapping ( World Health Organization, 2000). In each village, 100 randomly selected adults (≥15 years) were studied for LF infection and podoconiosis-related morbidity. In total 141,238 individuals from 1,442 communities in 775 districts were included in the study.

Trained health professionals conducted the survey; lymphoedema was diagnosed clinically by history and physical examination, and cases were tested for LF using a point-of-care test. Participants were requested to provide a finger-prick blood sample, which was tested for circulating *Wuchereria bancrofti* antigen using an immunochromatographic card test (ICT). For individuals with lymphoedema, an algorithm was used to identify possible differential diagnoses of podoconiosis ( Sime *et al.*, 2014). All individuals with ICT negative results underwent physical examination for signs and symptoms of podoconiosis. In this study, a confirmed podoconiosis case was defined as a person residing in the surveyed district for at least 10 years (to exclude cases who acquired the disease elsewhere) ( Kloos *et al.*, 1992), with lymphoedema of the lower limb present for more than 3 months, excluding other potential causes of lymphoedema( Sime *et al.*, 2014). Geographic coordinates of the sampled communities were recorded. 

We used information from the available community data (sample size and number of positive cases) at known locations (longitude and latitude) with a selection of environmental and socio-demographic datasets, to produce a gridded map of predicted prevalence of podoconiosis by implementing a geostatistical modelling approach.

***Covariates.*** A suite of remote sensing derived datasets was selected based on previous modelling work ( Deribe *et al.*, 2015b). The relationship between the following covariates and podoconiosis prevalence was explored: elevation and derived slope; long-term average of precipitation; enhanced vegetation index (EVI); clay and silt content of the top soil (0–15 cm), and night light-emissivity (see Supplementary File 1).

The elevation dataset was derived from a gridded digital elevation model produced by the Shuttle Radar Topography Mission ( http://srtm.csi.cgiar.org) ( Farr & Kobrick, 2000) and subsequently resampled to 1 km ^2^ resolution to match the resolution of the other datasets. The elevation surface was processed to obtain slope in degrees. The gridded precipitation layer was downloaded from the WorldClim database. The WorldClim database provides a set of global climate layers obtained by interpolation of precipitation data for the period 1950–2000 collected from weather stations distributed across the world ( Hijmans *et al.*, 2005). A raster surface of averaged EVI for the period 2000–2015 was obtained from the African Soil Information System (AfSIS) project ( http://africasoils.net/services/data/remote-sensing/land/). Soil data (clay and silt content at the top soil) were downloaded from the ISRIC-World Soil Information project included in the Harmonized Soil Map of the World ( http://www.isric.org/explore/isric-soil-data-hub). These datasets were obtained at 250m ^2^ and were subsequently resampled using bilinear interpolation to match the spatial resolution of the other gridded datasets (1 km ^2^). Finally, night-light emissivity captured by the Operational Linescan System instrument on board a satellite of the Defence Meteorological Satellite Programme was used as a proxy measure of poverty across Ethiopia ( https://ngdc.noaa.gov/eog/dmsp/downloadV4composites.html, Elvidge *et al.*, 1996). This instrument measures visible and infrared radiation emitted at night-time, resulting in remote imagery of lights on the ground. The brightness of light pixels varies on an arbitrary scale from 0–63 units, with the largest, well-electrified, urban areas yielding the highest values. This information has been correlated with gross domestic product in developed countries ( Doll *et al.*, 2006; Ebener *et al.*, 2005) and, although far from precise, would provide an indirect measure of poverty in developing countries ( Noor *et al.*, 2008). The major advantage of this dataset is that it can be obtained in a gridded continuous format at 1 km ^2^ resolution and by year since 1992. We considered the night-light emissivity measured in 2011 as a midpoint between the years of data collection.

Survey and covariate data were linked in ArcGIS 10.3 (Environmental Systems Research Institute Inc. [ESRI] Inc., Redlands CA, USA) based on the WGS-1984 Web Mercator projection at 1 km ^2^ resolution. Input grids were resampled, when necessary, to a common spatial resolution of 1km ^2^ using a nearest neighbour approach, clipped to match the geographic extent and aligned to a land mask template of Ethiopia.

### Geostatistical analysis

Empirical data and spatially matched covariates were used within a geostatistical framework. We developed a spatially explicit logistic regression model to predict podoconiosis prevalence at village level across Ethiopia. In the model, podoconiosis risk depended on the most relevant environmental risk factors, namely those most strongly associated with podoconiosis prevalence, as described above. Let *p(x)* denote the prevalence of podoconiosis at location *x*; the linear predictor for the log-odds is

$log\left\{\frac{p(x)}{1-p(x)}\right\}$ = *β*_0_ + *β*_1_ *Rainfall*( *x*) + *β*_2_ *Night Light Emissivity*( *x*) + *β*_3_ *Slope*( *x*) + *β*_4_ *EVI*( *x*) + *β*_5_ *Elevation*( *x*) + *β*_6_ *Elevation* ^2^( *x*) + *β*_5_ *Silt*( *x*) + *β*_6_ *Clay*( *x*) + *S*( *x*) + *Z*( *x*)

where *S(x)* are spatial random effects that account for spatial variation in podoconiosis prevalence between communities, not explained by the predictors, and Z *(x)* are unstructured random effects, also known as “nugget effect”, which capture extra-binomial variation within communities. For example, such differences might be due to individual variability (e.g. genetic predisposition or behavioural traits). More details on the model formulation of the geostatistical model can be found in Supplementary File 1.

We carried out validation of the model using a variogram-based procedure, which tested the compatibility of the adopted spatial structure with the data. More details are provided in Supplementary File 1. The model parameters were estimated by Monte Carlo maximum likelihood using the R 3.4.1 version package *PrevMap*, which implements parameter estimation and spatial prediction of generalized linear geostatistical models ( Giorgi & Diggle, 2017). The final fitted model was applied to produce continuous predictions of prevalence of podoconiosis among adults (≥15 years) at 1 km ^2^ resolution. A probability map of areas exceeding 1% prevalence (the threshold used to define podoconiosis endemicity) was also developed ( Deribe *et al.*, 2015d).

We used gridded maps of population density and age structure, obtained from the WorldPop project ( Linard *et al.*, 2012; Tatem *et al.*, 2007), to estimate the number of podoconiosis cases among the adult population. The map of predicted prevalence of podoconiosis was multiplied with the corresponding gridded map of estimated adult population resampled at the same spatial resolution. Using the resulting gridded map, the aggregate number of adults with podoconiosis at different administrative levels was extracted.

### Model uncertainty and validation statistics

We checked the validity of the assumed covariance model for spatial correlation using the Monte Carlo algorithm and empirical semi-variogram as described in Supplementary File 1. Additionally, a map of the number of standard errors from the posterior mean prevalence of podoconiosis was used to estimate the lower and upper confidence intervals of the predicted case estimates. Confidence interval maps determined the uncertainty regarding the estimated number of cases of podoconiosis.

## Results

### Data availability for podoconiosis

**Figure 1.**
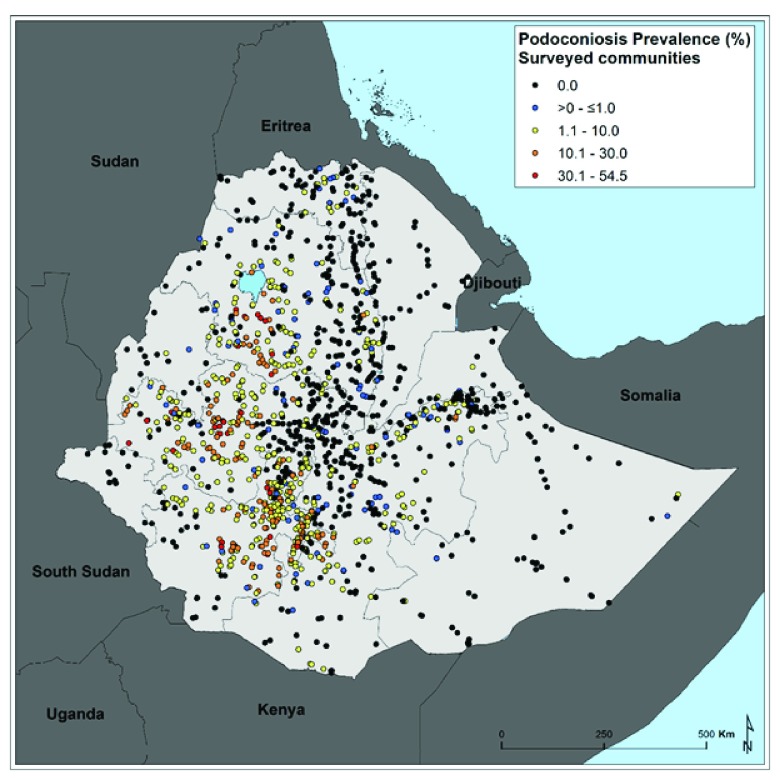
Distribution of 1,442 communities surveyed and prevalence of podoconiosis among adults (≥15 years).

Data included in this analysis are summarized in Table 1. In total, we identified 1,442 communities from unique locations in 775 districts. In 41.9% of surveyed districts, no cases of podoconiosis were identified. The distance between communities ranges from about 1 km to about 1,450 km. We have data from the nine Regional States and two City Administrative Councils of Ethiopia resulting in 141,238 individuals surveyed. Most of the surveyed communities (77%) were conducted in three Regional States: Amhara, Oromia and SNNP. Only Addis Ababa had fewer than 10 data-points. Overall, 91% of the community survey data were from the 2013 mapping study. The mean number of people examined per village was 97.6; the majority of communities (1,350, 93.6%) had more than 90 examined individuals. Figure 1 shows the spatial distribution of surveyed communities and podoconiosis prevalence at each site.

**Table 1.** Number of districts and communities surveyed, by region.

Region	Districts surveyed	Number of communities
Addis Ababa	4	8
Affar	32	64
Amhara	144	285
Benishangul Gumuz	20	21
Dire Dawa	7	14
Gambella	11	16
Harari	9	18
Oromia	298	541
SNNP	155	285
Somali	49	99
Tigray	46	91
**Total**	**775**	**1,442**

### Environmental factors associated with podoconiosis prevalence

**Figure 2.**
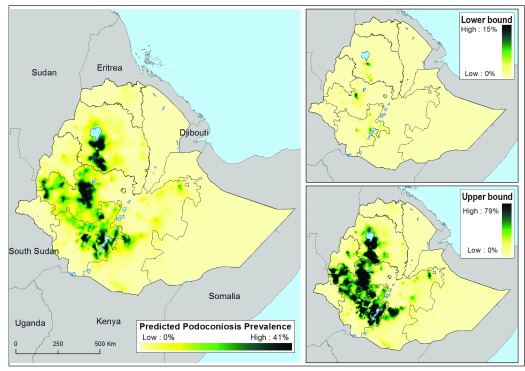
Predicted podoconiosis prevalence maps of Ethiopia; mean predicted prevalence and confidence intervals (95% CI).

Seven environmental variables, which were previously identified as potential predictors for podoconiosis in Ethiopia ( Deribe *et al.*, 2015b), were used to model podoconiosis prevalence. These included elevation and derived slope, annual precipitation, EVI, clay and silt soil content, and night-light emissivity (see Supplementary File 1). A geostatistical model with an isotropic and exponential spatial covariance function was fitted with the seven covariates and then used to predict podoconiosis prevalence for unsurveyed areas ( Figure 2).

### Estimation of podoconiosis cases across Ethiopia

**Figure 3.**
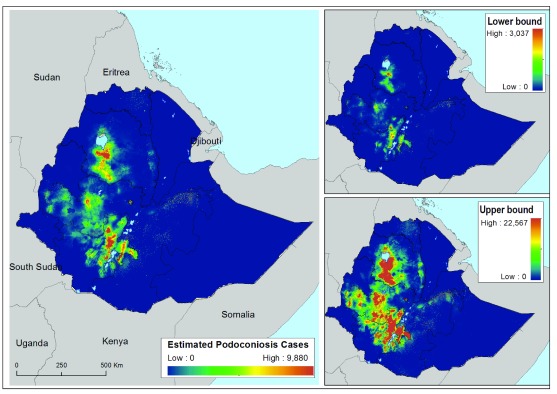
Estimated number of people with podoconiosis across Ethiopia: estimated number of cases and confidence intervals (95% CI).

Figure 2 and Figure 3 show the predicted podoconiosis prevalence in adults and the distribution of people with podoconiosis (i.e. estimates of podoconiosis cases), respectively. Nationally, our estimates suggest that 1,537,968 adults (95% Confidence Interval [CI], 290,923- 4,577,031) were living with podoconiosis in 2015 in Ethiopia. Three large regions (SNNP, Oromia and Amhara) contributed 99% of the cases. The highest proportion of all individuals with podoconiosis resided in the SNNP (39%), while 32% and 29% of people with podoconiosis resided in Oromia and Amhara Regional States, respectively. Tigray and Beneshangul Gumuz Regional States contributed marginally to the total number of people with podoconiosis, but there were almost no cases in the other regions ( Table 2).

**Table 2.** Estimated number of podoconiosis cases among adults in Ethiopia in 2015.

Region	Adult estimated cases	Lower Bound	Upper Bound	Population living in areas exceeding 1% podoconiosis prevalence
Addis Ababa	256	33	930	-
Affar	36	3	146	-
Amhara	446,858	77,486	1,331,010	8,852,685
Benishangul Gumuz	6,400	616	26,158	214,750
Dire Dawa	1	0	2	5,976
Gambella	201	15	902	11,841
Harari	8	1	32	-
Oromia	484,014	77,672	1,571,426	13,447,490
SNNP	598,676	134,920	1,640,511	13,580,281
Somali	61	2	354	4,860
Tigray	1,452	173	5,561	75,102
**Total**	**1,537,963**	**290,923**	**4,577,031**	**36,192,985**

Within regions, the distribution of podoconiosis cases is quite heterogeneous. In Amhara region, the majority of the cases were predicted to occur in East Gojam, West Gojam and South Gondar zones. In Oromia region, most podoconiosis cases were predicted to come from East and West Wellega, Illu Aba Bora and Jimma zones. In the SNNP region, Gamo Gofa, Hadiya, Sidama and Wolayita zones were predicted to be the most affected.

### Probability contour map and model validation

**Figure 4.**
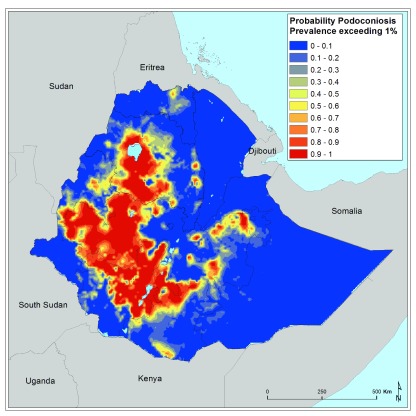
Map of probability of exceeding 1% podoconiosis prevalence.

We also estimated the continuous probability of exceeding 1% podoconiosis prevalence (the threshold considered for intervention) across the endemic areas ( Figure 4). The population living in areas that require intervention was estimated using the map. Overall, we estimated that 36 million people live in areas where the probability of exceeding 1% prevalence is greater than 75% ( Table 2).

The estimated podoconiosis prevalence was associated with varying degrees of uncertainty. The model was validated using the Monte Carlo simulation based on the fitted model and computing an empirical semi-variogram with the residuals, obtained by fitting a standard logistic regression with simulated data and predictors (see Supplementary File 1). We concluded that the adopted covariance model was compatible with the data, as the empirical semi-variogram fell within the 95% tolerance intervals computed via Monte Carlo simulation and a spatial correlation test of residuals.

## Discussion

Estimating the exact number of podoconiosis cases is difficult, due to a dearth of reliable epidemiological data and the absence of point-of-care diagnosis for podoconiosis. Data on the number of people with podoconiosis remain scarce in Ethiopia partly because of the challenges of definition and data collection. Here, we have collated up-to-date and reliable epidemiological data and developed a robust model to generate detailed national and subnational estimates for a given year.

Our analysis gives an estimate of 1,537,963 podoconiosis cases in Ethiopia in 2015. This figure is substantially higher than the previous estimate of 550,000 cases in 1974 ( Price, 1974). Nonetheless, the 1974 estimate is within the confidence interval of our estimates. It is unclear whether this difference is because of a sharp increase in the absolute number of cases due to neglect, or previous underestimation, or simply dramatic population growth (the Ethiopian population has increased fourfold since 1974) ( Federal Democratic Republic of Ethiopia Ministry of Health, 2016; Price, 1974). Podoconiosis has been recognised only recently by the health system, since it was first reported in the country in 1969. The scarcity of allocated resources, low prioritization, and poor understanding of the disease among programme planners and health care providers, have been the biggest obstacles to podoconiosis control in Ethiopia over the last four decades. The methods used to assess the number of people with podoconiosis have changed since 1974 ( Deribe *et al.*, 2015b), when previous work by Price was sustained by market surveys ( Oomen, 1969; Price, 1974). In recent years, community-based surveys with full clinical diagnosis and diagnostic tests to exclude other causes of elephantiasis have been used ( Deribe *et al.*, 2015a; Deribe *et al.*, 2015b; Sime *et al.*, 2014). The availability of better differential diagnosis and assessment methods are also contributing factors to the difference in the number of estimated cases. 

Ninety-nine percent of podoconiosis cases were estimated to occur in the three largest Regional States (Amhara, Oromia and SNNP) of Ethiopia, which is consistent with previous prevalence studies. For example, a 2007 study conducted in SNNP estimated the prevalence of podoconiosis in Wolayita zone to be 5.46% (approximately 81,000 cases) ( Desta *et al.*, 2003; Tekola *et al.*, 2006). Our model estimates that 72,723 people are predicted to be suffering from podoconiosis in Wolayita zone, with the 2007 estimate falling within the 95% confidence interval of our prediction.

Our results clearly reflect the magnitude of the problem for the Ethiopian public health system. The number of people living with podoconiosis is unacceptably high and indicates the need for rapidly increased coverage with interventions. Our model and the derived estimates have been produced at a high spatial resolution (1 km ^2^), which will enable planning at lower administrative levels such as the health facility catchment zone or district. This level of planning will increase the efficiency, effectiveness and scale-up of prevention and morbidity management plans. These results will influence prioritization of geographical areas based on caseloads, and will guide local health facilities to strengthen surveillance to detect new cases. 

The predicted probability that the local prevalence of podoconiosis exceeds 1% (the threshold to define endemic areas) ( Deribe *et al.*, 2015c; Deribe *et al.*, 2015d) in most of the surveyed areas is clearly demarcated. There appears to be little uncertainty in the map ( Figure 4), as the probability of the prevalence exceeds the threshold in most areas is either very high (>0.9) or very low (<0.1). However, in a few areas, such as north and south Wello, the results are less clear. In such areas, it will be important to inspect the available data in detail to assess the operational implications for local prevention and morbidity management interventions. Future research will aim to extend to the current analysis to account for individual risk factors of podoconiosis. This will then allow us to generate maps of exceedance probability under different individual risk profiles.

Our analysis has, for the first time, enabled detailed subnational estimation of podoconiosis in Ethiopia. We used nationwide survey data to estimate the number of cases and remotely sensed data to model the prevalence of podoconiosis. Nonetheless, it is important to consider limitations in the interpretation of results and their future applications. First, there were regions with limited data; Addis Ababa and Dire Dawa City Administrations had few communities surveyed. However, the two City Administrations accounted for only 4% of the national population and 0.34% of the estimated 35 million people at risk of podoconiosis in Ethiopia ( Deribe *et al.*, 2015b). Second, we included several sets of spatial covariates, but the covariates included do not represent all the potential drivers of podoconiosis. For example, our model did not account for the use of protective measures such as frequency of shoe wearing, because this information is not available in a continuous gridded format. We tried to minimize this limitation by including a proxy measure of poverty (night-light emissivity). As these covariates become available future studies should incorporate them. Third, the confidence interval of our estimation is very wide, which implies there are factors not accounted for in our modelling that determine the distribution of podoconiosis. Potentially these could be related to individual behaviours (e.g. shoe wearing and foot hygiene) ( Deribe *et al.*, 2015a; Molla *et al.*, 2013) or more detailed soil characteristics ( Molla *et al.*, 2014). Our modelling included important soil characteristics that might drive the distribution of podoconiosis; however, detailed soil characteristics including mineral contents (such as smectite, quartz and mica) ( Molla *et al.*, 2014) found to be associated with podoconiosis distribution, were not available in a high‐resolution spatial data or layers. Therefore, our estimates of podoconiosis cases should be interpreted with caution given the wide confidence interval. Nonetheless, it is important to note that our maps on the probability that the local prevalence of podoconiosis exceeds 1% ( Deribe *et al.*, 2015d) are of high resolution and can serve as an important input for programme planning and decision making.

Our analysis provides a framework for modelling podoconiosis distribution in Africa and globally. Building on the current work, we will model the distribution of podoconiosis in other highly endemic countries that are generating national data ( Deribe *et al.*, 2017). The availability of nationally representative data is important for such work, such as the integration of podoconiosis surveys with other ongoing surveys. Endemic countries should also be strengthening the routine surveillance of podoconiosis cases to generate sustainable and more representative data ( Deribe *et al.*, 2017). The current work also provides important data for estimating the burden of podoconiosis in terms of disability-adjusted life-years (DALYs) ( Murray *et al.*, 2012; Salomon *et al.*, 2015), using the well-established disability weightings for lymphoedema generated by the Institute for Health Metrics and Evaluation ( Murray *et al.*, 2012; Salomon *et al.*, 2015). 

This high-resolution mapping of podoconiosis has enabled us to better estimate the number of cases in Ethiopia. The estimate presented here clearly shows that podoconiosis is highly endemic throughout Ethiopia and the number of cases over the last 40 years has remained unacceptably high. While we now have a good estimate of the number of people with podoconiosis, it is important to also understand its burden in terms of DALYs and economic and productivity losses ( Deribe *et al.*, 2015d). Given the high profile of podoconiosis on the global health agenda and the presence of low cost prevention, and morbidity management and disability prevention services, it is our collective responsibility to scale-up interventions rapidly. 

### Data availability

The data used in this study is generated through a collaboration between the Ethiopian Public Health Institute (EPHI), Addis Ababa University and Brighton and Sussex Medical School (BSMS). All data sharing requests are reviewed and approved by them. To initiate the data access process, please contact the BSMS Research Governance and Ethic Committee: rgec@bsms.ac.uk, who will provide guidance for accessing the data.

Access to data for covariates are detailed in the Methods.

## Supplementary material

**Supplementary File 1: Geostatistical analysis.** The supplementary file provides maps of the covariates, the model formulation and validation.
