# Supplementary material for: Estimating the number of cases of podoconiosis in Ethiopia using geostatistical methods
Source: Wellcome Open Res. 2017 Dec 15;2:78. Originally published 2017 Sep 4. [Version 2] doi: 10.12688/wellcomeopenres.12483.2 (PMC5668927; doi:10.12688/wellcomeopenres.12483.2)
Supplement: Supplementary file 1 [file wellcomeopenres-2-14630-s0001.tgz › 93055e7c-4dc4-4c32-a4ef-94314de55f97.pdf]

Estimating the number of cases of podoconiosis in Ethiopia using geostatistical methods

Supplementary material - Geostatistical analysis

December 11, 2017

Covariates

Seven covariates were used to model the spatial variation in podoconiosis prevalence: elevation and derived slope, long-term average of precipitation, enhanced vegetation index (EVI), clay and silt content at the top soil (0-15 cm) and night light-emissivity. Each of these is displayed in Figure 1.

Model formulation

Let Y_i denote the random variable associated with the number of podoconiosis cases, out of n_i individuals, sampled at a village location x_i in Ethiopia. We assume that conditionally on spatial random effects, $S(x_i)$, and Gaussian noise, $Z(x_i)$, the Y_i are mutually independent binomial with probability of a positive test for podoconiosis, p_i. The linear predictor is

$$\log\left\{\frac{p_i}{1-p_i}\right\} = d(x_i)^\top \beta + S(x_i) + Z(x_i), \tag{1}$$

where $d(x_i)$ is a vector of explanatory variables with associated vectors of regression coefficients β. A plot of the logit-transformed prevalence against elevation, shows a positive association up to about 2000 meters and negative thereafter (Figure 2). For this reason elevation was included in the model using a quadratic polynomial. The spatial random effects $S(x)$ can be interpreted as the cumulative effect of unmeasured risk factors for podoconiosis. Finally, the unstructured random effects $Z(x)$ represents extra-binomial variation within villages.

We assume that $S(x)$ is a stationary and isotropic Guassian process with mean zero and covariance function given by

$$\text{cov}\{S(x), S(x')\} = \sigma^2 \exp\{-\|x - x'\|/\phi\},$$

where $\|x - x'\|$ is the Euclidean distance between any two locations x and x'.

We use τ^2 to denote the variance of the Gaussian noise $Z(x)$.

We use Monte Carlo maximum likelihood (MCML) (Geyer & Thompson, 1992; Geyer, 1994, 1996, 1999) to obtain point estimates of β, σ^2, ϕ and τ^2.

Table 1 reports the (MCML) and the corresponding 95% confidence intervals.

Table 1: Monte Carlo maximum likelihood estiamtes with association 95% confidence intervals (CI).

	Estimate	95% CI
Intercept	-13.931	(-14.944, -12.917)
Precipitation $\times 10^3$	1.268	(0.475, 2.060)
Night emissivity lights	-0.006	(-0.019, 0.006)
Slope	-0.024	(-0.051, 0.002)
EVI	0.871	(-0.504, 2.245)
Elevation $\times 10^3$ (linear term)	7.376	(6.271, 8.481)
Elevation $\times 10^3$ (quadratic term)	-1.999	(-2.261, -1.738)
Silt	0.035	(0.017, 0.052)
Clay	0.003	(-0.003, 0.009)
$\log(\sigma^2)$	0.979	(0.774, 1.185)
$\log(\phi)$	3.928	(3.657, 4.199)
$\log(\tau^2)$	-0.857	(-1.325, -0.390)

Model validation

We check the validity of the assumed covariance model for the spatial correlation using the following Monte Carlo algorithm.

1. Simulate $S(x_i)$ and $Z(x_i)$ under the fitted model at each of the sampled village locations x_i.

2. Simulate binomial data y_i based on (1).

3. Fit a standard logistic regression (i.e. $S(x_i) = Z(x_i) = 0$, for all x_i) to the simulated data y_i using explanatory variables $d(x_i)$.

4. Obtain the Pearson's residuals from the standard logistic regression of the previous step and compute the empirical semi-variogram.

5. Repeat steps 1 to 4 for 10,000 times.

6. Use the resulting 10,000 empirical semi-variograms to compute 95% tolerance intervals at each distance bin.

7. Compute the empirical semi-variogram using the residuals of a standard logistic regression as in step 3, for the observed data.

8. If the empirical semi-variogram from step 7 falls inside the 95% tolerance intervals, we conclude that the adopted covariance function is compatible with data. If, instead, the empirical semi-variogram from step 7 falls outside the 95% tolerance intervals, we conclude that the assumed covariance function is not compatible with the data.

Figure 3 shows the results of the outlined validation procedure. Since the empirical semi-variogram (solid line) falls within the 95% tolerance intervals (dashed lines), we then conclude that the adopted covariance model is compatible with the data.

References

Geyer, C. J. (1994). On the convergence of Monte Carlo maximum likelihood calculations. *Journal of the Royal Statistical Society, Series B* **56**, 261–274.

Geyer, C. J. (1996). Estimation and optimization of functions. In *Markov Chain Monte Carlo in Practice*, W. Gilks, S. Richardson & D. Spiegelhalter, eds. London: Chapman and Hall, pp. 241–258.

Geyer, C. J. (1999). Likelihood inference for spatial point processes. In *Stochastic Geometry, Likelihood and Computation*, O. E. Barndorff-Nielsen, W. S.Kendall & M. N. M. van Lieshout, eds. Boca Raton, FL: Chapman and Hall/CRC, pp. 79–140.

Geyer, C. J. & Thompson, E. A. (1992). Constrained Monte Carlo maximum likelihood for dependent data. *Journal of the Royal Statistical Society, Series B* **54**, 657–699.

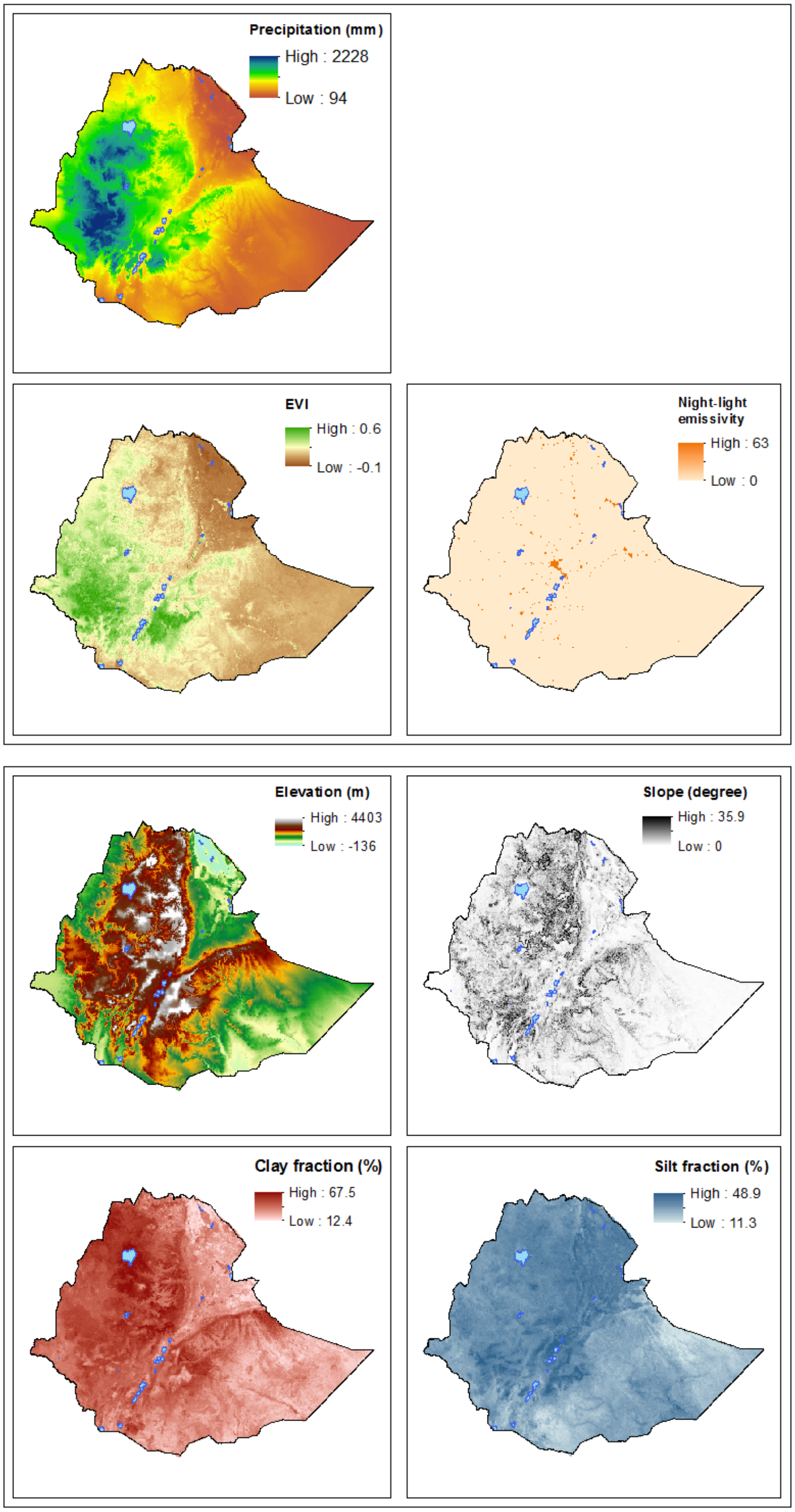

Figure 1: Spatial covariates used to model podocnoiosis prevalence.

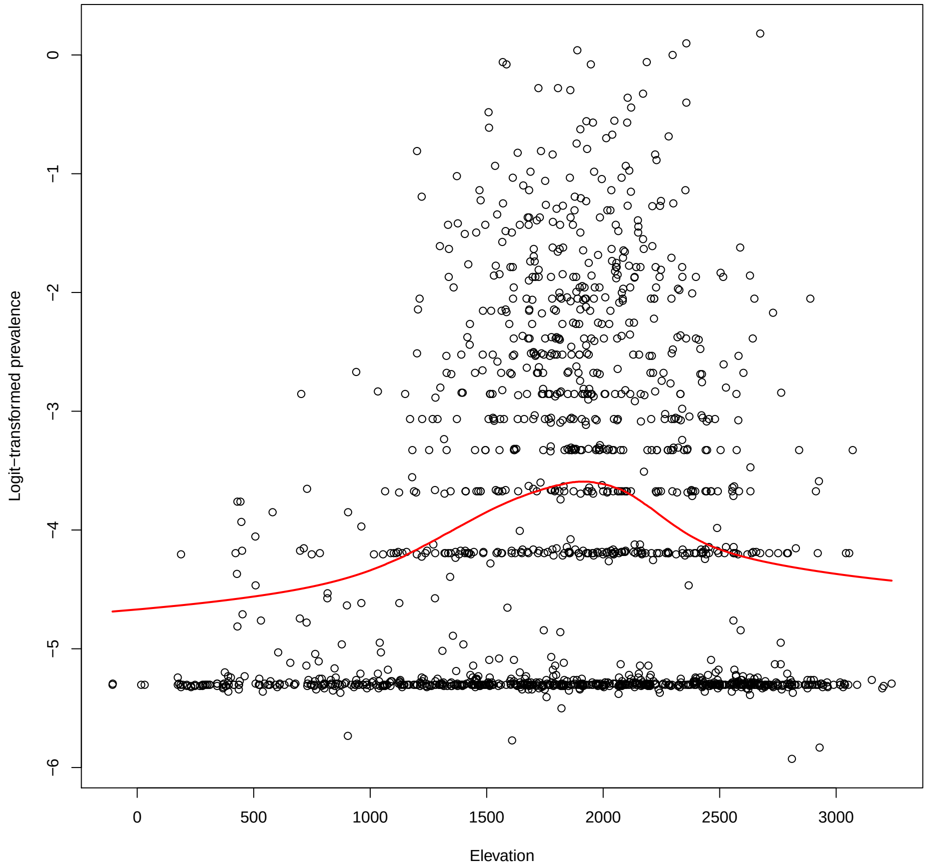

Figure 2: Plot of the logit-transformed prevalence against elevation. The red line is obtained using locally weighted smoothing.

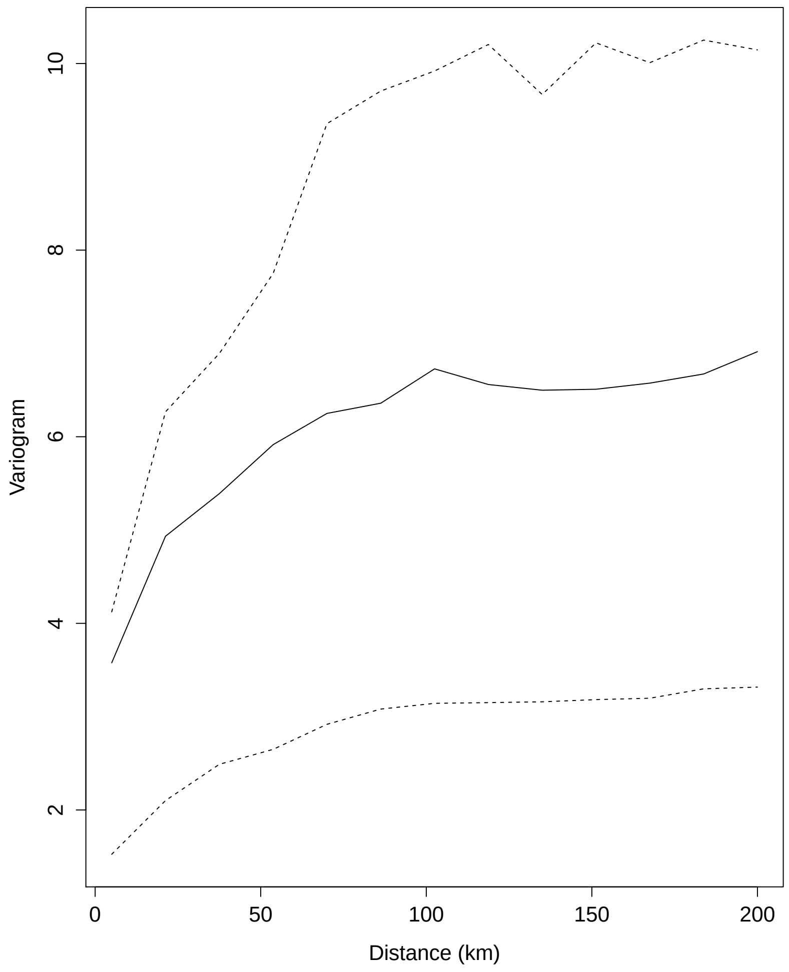

Figure 3: Results from the model validation. The dashed lines represent the 95% tolerance intervals from step 6. The solid line corresponds to the observed semi-variogram of step 7.
