# Supplementary material for: Estimating the number of cases of podoconiosis in Ethiopia using geostatistical methods
Source: Wellcome Open Res. 2017 Dec 15;2:78. Originally published 2017 Sep 4. [Version 2] doi: 10.12688/wellcomeopenres.12483.2 (PMC5668927; doi:10.12688/wellcomeopenres.12483.2)
Supplement: Peer review history

# Peer review: Referee response for version 2

**DOI:** 10.21956/wellcomeopenres.14630.r29087

**Published:** 2018-01-12

**Authors:** Michelle C. Stanton

**Affiliations:** 1 Department of Parasitology, Liverpool School of Tropical Medicine, Liverpool, UK; 2 Lancaster Medical School, Lancaster University, Lancaster, UK

Thank you to the authors for the edits made. I am now satisfied with this article and I am happy to approve it for publication.

I have read this submission. I believe that I have an appropriate level of expertise to confirm that it is of an acceptable scientific standard.

# Peer review: Referee response for version 1

**DOI:** 10.21956/wellcomeopenres.13516.r26136

**Published:** 2017-11-06

**Authors:** Delenasaw Yewhalaw

**Affiliations:** 1 Tropical and Infectious Diseases Research Center, Jimma University, Jimma, Ethiopia

**Minor essential comments**

 This paper assessed the prevalence and estimated number of cases of podoconiosis in Ethiopia. It is an important work which provides input to health decision making and planning process for podoconosis intervention and elimination. This study moreover estimates the number of podoconiosis cases and prevalence at sub-country/sub-regional level such an approach is important for countries like Ethiopia which initiated elimination agenda and where morbidity, disease occurrence and severity varies from one area to the other in the country.

**Introduction**:

 OK

**Methodology**:

 The methodology is sound and clear. However, it would have been good if authors also present additional climatological data such as temperature and relative humidity which have an impact on EVI.

 Authors performed the analysis based on data collected from 2008 to 2013. Why were authors interested to estimate number of podoconiosis cases and prevalence for 2015? Authors should justify why they didn’t estimate for 2014? or from 2014-2016 to see the trend and distribution? This could also be important to evaluate the impact of intervention on the burden of podoconiosis at local and national level.

**Results**:

 There was wide confidence interval for both estimated number of podoconocis cases and its prevalence in each surveyed region. This shows that the authors didn’t take into account important predictors as it has also been mentioned by the authors or because of the problem of model selection. This could have implication on resource mobilization for disease prevention, morbidity management or elimination efforts.

**Discussion**:

 OK

**Conclusion** **:**

 OK

I have read this submission. I believe that I have an appropriate level of expertise to confirm that it is of an acceptable scientific standard.

# Response

**Published:** 2017-12-12

**Authors:** Kebede Deribe

**Affiliations:** Wellcome Trust Brighton and Sussex Center for Global Health Research, Brighton and Sussex Medical School, UK

Many thanks for your thoughtful comments. As you rightly indicated the study used data from two surveys conducted between 2008 and 2013. We have estimated the number of podoconiosis cases for the years 2015 because we have raster data of population density data for Ethiopia for 2010, 2015 and 2020. The closest year to our estimates and the current period would be 2015. Given the chronic nature of podoconiosis we do not expect significant incident cases to affect our estimates significantly. Nonetheless we take your point of the need for updating the estimates and figures for the years to come as more epidemiological data and covariates become available.

We have acknowledged and discussed the potential reasons for wide confidence interval.

Multilevel factors are involved in podoconiosis risk and diseases progression (individuals, household and environmental). Individual factors such as genetic susceptibility and footwear use are not available at the format and resolution (continuous gridded format) required for such geostatistical analysis. Given the limitation, nonetheless, our estimates provide important information for planning and monitoring progress in the control and elimination of podoconiosis. Temperature and relative humidity have been considered in our previous analysis and were excluded because of multicollinearity with other covariates and therefore excluded from the analysis.             

  

Thank you again for reviewing our manuscript.

# Peer review: Referee response for version 1

**DOI:** 10.21956/wellcomeopenres.13516.r26135

**Published:** 2017-11-01

**Authors:** Henok Tadesse Ayele

**Affiliations:** 1 McGill University, Montreal, QC, Canada

The authors investigated the prevalence and associated environmental factors of Podoconiosis, which is a disease of high public health importance. Their findings have paramount programmatic implications. Overall, this is a well-written manuscript; however, I have some concerns, which are listed below.

 Major concerns 

1. The authors have not adequately mentioned the details of sample size determination. It is not clear whether the WHO definition of village (50X50km grid) apply in their sampling clusters (What is village?) and the WHO study was assessing the prevalence of filarial elephantiasis (how valid to assume the sampling technique is similar for these two diseases and climate stay constant in the last 30 years?). Did they take the clustering effect in to consideration?
  Their previous study ^1^ reported that two kebele (smallest administrative units) were selected from each districts based on reported history of lymphoedema cases collected through interviewing the woreda health officials, health providers, and village leaders 1 day before the survey. How valid is the selection of village with known cases? The potential selection bias might overestimate the prevalence.
2. The model does not look optimally fitted. It only includes environmental factors (individual study participants characteristics were not included). Night light emissivity was assumed to capture individual participants’ characteristics as a proxy measure but not sure, if it is valid assumption. Moreover, from your previous study mean annual land surface temperature and altitude were strong environmental predictors of podoconiosis. Yet they are excluded from the current prediction model.
3. As a follow up of no. 2 what methods you used to select the predictors finally fitted to yield a parsimonious model. 
4. A geostatistical model was used to estimate prevalence of podoconiosis in non-surveyed area but the size of non-surveyed area is not mentioned. How comparable were the surveyed and unsurveyed areas (A purposive sampling was used to select the villages rather than using the random sampling). Do you have data (on seven environmental factors) in non-surveyed areas?
5. The study might have benefited from a multivariate Bayesian generalized linear spatial model since the proper analysis seems a multivariate Bayesian generalized linear spatial modeling (Slater 2013) which would fit both environmental and individual participant’s data. You might probably mention this in the discussion part.

Minor concerns

1. The total population size was frequently cited from the FMOH even though the primary source is Central Statistical Agency.
2. I would mention the coverage measure of prediction model (C statistics for example).

I have read this submission. I believe that I have an appropriate level of expertise to confirm that it is of an acceptable scientific standard.

## References

[1] DeribeKBrookerSJPullanRLSimeHGebretsadikAAssefaAKebedeAHailuARebolloMPShafiOBockarieMJAseffaAReithingerRCanoJEnquselassieFNewportMJDaveyG: Epidemiology and individual, household and geographical risk factors of podoconiosis in Ethiopia: results from the first nationwide mapping. *Am J Trop Med Hyg*.2015;92(1) : 10.4269/ajtmh.14-0446 148-58 10.4269/ajtmh.14-0446 25404069PMC4288951

# Response

**Published:** 2017-12-12

**Authors:** Kebede Deribe

**Affiliations:** Wellcome Trust Brighton and Sussex Center for Global Health Research, Brighton and Sussex Medical School, UK

Thanks for taking the time to review our manuscript. We have provided the summary of the response to your comments. The WHO guidelines for lymphatic filariasis (LF) mapping recommend the selection of two villages per district. We have adopted this for integrated mapping of LF and podoconiosis, as detailed in previous publication (Sime *et al. *2014). In our subsequent analysis we have demonstrated that podoconiosis has higher range of spatial dependence than LF (50X50km grid) (Deribe *et al.* 2015a).

We take communities as common name for administrative level next to *kebele*, this could be *“gote”,* which differs from region to region. The detailed sampling strategy used and their implications have been discussed in our previous publications (Deribe *et al. *2015a, Sime *et al.* 2014). In the current model we have included seven covariates which have shown to be associated with podoconiosis in Ethiopia; clay fraction, silt fraction, elevation (altitude), slope, distance from closest waterway, Enhanced Vegetation Index [EVI]) and precipitation (Deribe et al. 2015a, Deribe et al. 2015b). The covariates were available as gridded maps and therefore suitables for being used eventually for projection on unsampled areas. Mean annual land surface temperature showed multicollinearity with other variable and was excluded from the analysis (Deribe *et al.* 2015a, Deribe *et al.* 2015b). We decided to include night-light emissivity as a proxy of poverty, as it’s detailed in methods section in the manuscript. Previous studies have demonstrated that night light emissivity can be associated with household consumption, assets and poverty (Jean *et al.* 2016).

We have acknowledged in the manuscript that the lack of individual and household covariates at the spatial resolution of our analysis is one of the limitation of our study, what would explain the wide confidence interval in the model prediction. As we replied to another reviewer, unfortunately, individual factors such as genetic predisposition and footwear use, key factors for the occurrence of podoconiosis, are not available in a suitable format for making projections (continuous gridded format).

In the discussion, we have acknowledged that one of the possible improvements of the current analysis would be the inclusion of individual-level risk factors. However Slater and Michael (Slater and Michael 2013) developed a geostatistical model for LF that did not differ from our approach; also, they did not include individual-level variables to fit final model. Their model differs from ours in the modelling approach implemented, they used Bayesian inference for fitting a geostatistical model whereas we used a likelihood-based approach. In this regard, we do not expect any tangible difference in our predictive inferences, had we used a Bayesian framework. Our approach pursues to maximize the likelihood function, thereby not making any assumptions on priors which may not be informative.

**References**

Deribe K, Brooker SJ, Pullan RL, Sime H, Gebretsadik A, Assefa A, Kebede A, Hailu A, Rebollo MP, Shafi O, Bockarie MJ, Aseffa A , Reithinger R, Cano J, Enquselassie F, Newport MJ and Davey G. 2015a. "Epidemiology and Individual, Household and Geographical Risk Factors of Podoconiosis in Ethiopia: Results from the First Nationwide Mapping." Am J Trop Med Hyg 92(1):148–58.

Deribe K, Cano J, Newport MJ, Golding N, Pullan RL, Sime H, Gebretsadik A, Assefa A, Kebede A, Hailu A, Rebollo MP, Shafi O, Bockarie MJ, Aseffa A, Hay SI, Reithinger R, Enquselassie F, Davey G and Brooker SJ. 2015b. "Mapping and Modelling the Geographical Distribution and Environmental Limits of Podoconiosis in Ethiopia." PLoS Negl Trop Dis 9(7):e0003946.

Jean N, Burke M, Xie M, Davis WM, Lobell DB and Ermon S. 2016. "Combining Satellite Imagery and Machine Learning to Predict Poverty." Science 353(6301):790.

Sime H, Deribe K, Assefa A, Newport MJ, Enquselassie F, Gebretsadik A, Kebede A, Hailu A, Shafi O, Aseffa A, Reithinger R, Brooker SJ, Pullan RL, Cano J, Meribo K, Pavluck A, Bockarie MJ, Rebollo MP and Davey G. 2014. "Integrated Mapping of Lymphatic Filariasis and Podoconiosis: Lessons Learnt from Ethiopia." Parasit Vectors 7(1):397.

Slater H and Michael E. 2013. "Mapping, Bayesian Geostatistical Analysis and Spatial Prediction of Lymphatic Filariasis Prevalence in Africa." PLoS One 8(8):e71574.

# Peer review: Referee response for version 1

**DOI:** 10.21956/wellcomeopenres.13516.r25655

**Published:** 2017-09-15

**Authors:** Bereket Yakob

**Affiliations:** 1 Department of Global Health and Population, Harvard T.H. Chan School of Public Health, Boston, MA, USA

**General**

 Overall, the manuscript is written well, has good scientific merit and high practical significance.

 Below raised are few points to better the paper.

**Abstract**

 Method: You collected most of the data in 2013 but you did not explicitly stated from where the rest of the data came. 

 Results: Define endemicity of podoconiosis. 

 Discussion: What are the low cost interventions? What do you mean by “our collective responsibility”?

**Introduction**

 Written well

**Methods**

 Please state if the data were collected from a point in time or repeated overtime from 2008 – 2013

 Did the figures in the results (magnitude and spatial distribution) refer to 2008/2013 or estimated for 2017? 

**Results**

**Data availability …**

 What are data points?

 What do you mean by unique locations in the districts? Please explain

**Estimation of podoconiosis cases**

 In the tables and figures, indicate to which year the data refer to (2008, 2013 or 2017). It’s not clear if they were estimations for 2008 and/or 2013 or projections for 2017

 Table 2: insert one column to show prevalence for each of the regions

**Discussion**

 Written well, and few queries are forwarded to better the section

 Please explain: Why was the magnitude podoconiosis very high in Amhara, Oromia and SNNP regions?

I have read this submission. I believe that I have an appropriate level of expertise to confirm that it is of an acceptable scientific standard.

## References

[1] DeribeKCanoJGiorgiEPigottDGoldingNPullanRNoorACromwellEOsgood‐ZimmermanAEnquselassieFHailuAMurrayCNewportMBrookerSHaySDaveyG: Estimating the number of cases of podoconiosis in Ethiopia using geostatistical methods. *Wellcome Open Research*.2017;2: 10.12688/wellcomeopenres.12483.1 10.12688/wellcomeopenres.12483.1 29152596PMC5668927

# Response

**Published:** 2017-12-12

**Authors:** Kebede Deribe

**Affiliations:** Wellcome Trust Brighton and Sussex Center for Global Health Research, Brighton and Sussex Medical School, UK

Many thanks for your comments. As it is indicated on the methods section we have used two cross-sectional datasets collected in 2008 and 2013. The data were collected from communities across the country from 775 districts. Podoconiosis endemicity has been defined as prevalence>1%, as it is stated in the methods section of the article. Based on the prevalence data and environmental covariates and using geostatistical modelling we estimated the number of cases of podoconiosis for the year 2015. As can be seen from the results, the magnitude of podoconiosis is estimated to be higher in Amhara, Oromia and SNNP Regional states of Ethiopia. These areas are environmentally more suitable for the occurrence of podoconiosis than others, as it has been demonstrated in a previous ecological modelling exercise (Deribe K *et al.* 2015b). The prevention of podoconiosis is rooted in promotion of regular footwear use and foot hygiene. The treatment is based on hygiene based morbidity management, as it has been discussed throughout the manuscript. By “collective responsibility” we mean the responsibility of podoconiosis elimination needs the involvement of multiple actors, the government, donors, program planner and implementers, that should act collectively to provide services to the people who need them.

**Reference**

Deribe K, Cano J, Newport MJ, Golding N, Pullan RL, Sime H, Gebretsadik A, Assefa A, Kebede A, Hailu A, Rebollo MP, Shafi O, Bockarie MJ, Aseffa A, Hay SI, Reithinger R, Enquselassie F, Davey G and Brooker SJ. 2015b. "Mapping and Modelling the Geographical Distribution and Environmental Limits of Podoconiosis in Ethiopia." PLoS Negl Trop Dis 9(7):e0003946.

# Peer review: Referee response for version 1

**DOI:** 10.21956/wellcomeopenres.13516.r25654

**Published:** 2017-09-13

**Authors:** Michelle C. Stanton

**Affiliations:** 1 Department of Parasitology, Liverpool School of Tropical Medicine, Liverpool, UK; 2 Lancaster Medical School, Lancaster University, Lancaster, UK

This article is well written and addresses an area of great public health importance in Ethiopia. There are numerous questions/comments about the data and methods used however which I have outlines below, broken down by section.

**Abstract:** Please provide a clearer idea of the burden of risk in the form of percentage of the population at risk in addition to absolute numbers

**Introduction: **Please provide a brief introduction to podoconiosis, how it’s transmitted and the currently implemented interventions

**Methods:**

- Please give a brief description of the rationale of why these covariates may be associated with podoconiosis risk.
- Please describe how elevation was resampled to 1km ^2^. What was the original spatial resolution of the data?
- Can averaged (modelled) rainfall over the period 1950-2000 be used to represent rainfall during the study period? What measure of rainfall was used? Total annual rainfall?
- Is average EVI over a 15 year period (2000-2015) a meaningful measure for this analysis? Were other summaries of EVI considered?
- What time period does the Harmonized Soil Map represent?
- What year was selected for the Night Light Emissivity data?
- In the equation for the geostatistical model, please clarify that NLE represents night light emissivity.
- How was the form of the covariate in the model decided upon e.g. elevation ^2^ is included in the model. Did you do any exploratory analysis prior to fitting the model?
- Were any other goodness of fit measures considered in addition to using the variogram-based procedure described e.g. some form of assessment of the predictive ability of the model using approaches such as cross-validation?
- Did you perform any model selection?

**Results:**

- Could you provide a sense of how far apart the surveyed villages were?
- You state that 91% of your data were collected in 2013. Given that you previously state that data is collected between 2008-2013, could you give an indication of the spread of data over these earlier years?
- First paragraph of the ‘Environmental factors associated with podoconiosis prevalence’ appears to repeat information that has already been given in the Methods. Please revise.
- You state that ‘the most parsimonious geostatistical models was fitted’. How did you establish parsimony given no model selection procedure has been described? Could you also include a table to describe the estimates of the parameters in the model, or minimally a description of the direction of their influence on prevalence. How big an impact does the spatial term S(x) have on the fitted model e.g. did the covariates describe most of the spatial variability or did S(x) play a big role?
- Could you use different colours in Figure 2 to represent the different scales, or adjust the maps so that they are all on the same scale. It is difficult to assess the amount of uncertainty in the estimates in its current form. Similarly could you adjust the colours/scale in Figure 3. Alternatively, have you considered producing a map of the width of the confidence interval instead of separate maps of the upper and lower bounds? You mention in the discussion that the confidence intervals are very wide, but this is difficult to discern from the current maps.
- Please indicate the proportion of the populations in each region that are at risk of podoconiosis as well as absolute numbers.
- As mentioned above, have you considered other methods of validating your model predictions in addition to determining that the adopted covariance model was compatible with the data? Some assessment of model fit/model predictions is needed.

**Discussion:**

- A comparison is made between the estimated podoconiosis cases obtained using this study and the number estimated in 1974. Given that the authors recognise that there’s been a dramatic change in the population since 1974, it is not appropriate to compare absolute numbers. A comparison of prevalence would be much more meaningful.

I have read this submission. I believe that I have an appropriate level of expertise to confirm that it is of an acceptable scientific standard, however I have significant reservations, as outlined above.

# Response

**Published:** 2017-12-12

**Authors:** Kebede Deribe

**Affiliations:** Wellcome Trust Brighton and Sussex Center for Global Health Research, Brighton and Sussex Medical School, UK

Many thanks for your important review. Please kindly see below the summary response we have given to each of your comments. In the current work our objective is to estimate the number of cases of podoconiosis. In a previous work we already estimated the population at risk and the results were published (Deribe K *et al.* 2015b). The current work is dedicated to estimating the number of cases of podoconiosis. To make the work in the context of our analysis we opted to use comparison of the number of cases. We hope given the clear contextual differences the readers can make the best out of our discussions and estimations. 

In the introduction part a brief paragraph about podoconiosis has been included **“**Podoconiosis is an endemic, non-filarial lymphoedema of the lower limb. It affects genetically susceptible individuals who often go barefoot (Price E 1990). It causes bilateral, often below knee lymphoedema of the lower limb.  Based on existing evidence, the most accepted cause of podoconiosis is that of mineral particle-induced inflammation on a background of genetic susceptibility(Price E 1990). Specific control methods include the use of footwear, regular foot hygiene and floor coverings. For those with the diseases management of the lymphoedema-related morbidity is recommended which includes foot hygiene, foot care, wound care, compression, exercises, elevation of the legs and treatment of acute infections (Deribe et al., 2015a).”

The selection of covariates is based on previous works that have been published elsewhere (Deribe K *et al.* 2015a, Deribe K *et al.* 2015b).  This has been stated in first paragraph of section describing the selected covariates. We obtained the elevation gridded dataset at 1km ^2^ from the CSI-CGIAR project ( http://srtm.csi.cgiar.org) (Farr TG and Kobrick M 2000), as we mention under methods. The elevation originally has been obtained by RADAR imagery at 30m resolution by the USGS and it has been resampled by CSI-CGIAR to make it manageable and usable for different purposes. We provide in the manuscript the link to the CSI-CGIAR where more details on data processing is given. The rainfall used in the current model was the mean annual rainfall. Podoconiosis is a chronic disease which is a result of long term exposure to red clay soil. Those with the disease may have developed through decades of exposure to the putative minerals. Therefore, the use of long term average of environmental covariates is justifiable. 

General composition of soils is not expected to vary significantly over time. The Harmonized Soil Map is the result of a robust modelling work over a large number of geo-referenced point data (100,000) available at the World Soil Information Service. This corresponds with over 5 million soil records. This project has been improving their model estimates based on increased number of sampled sites and applying more robust modelling approaches.  More details on the construction of this gridded maps of soil composition are provided in this reference(Hengl T et al. 2017). The version we have used in this project corresponds to the most updated version released in 2016. Night-light emissivity dataset correspond to year 2011, midpoint between years of data collection. We have included this information in the manuscript.

The equation for the geostatistical model has been revised. Now the equation reads:

We did carry out an initial exploratory analysis which showed a non-linear relationship between elevation and the empirical podoconiosis prevalence. More specifically, we found that the latter increased for increasing elevation up to about 2000 meters and decreased thereafter. For this reason elevation was included in the model using a quadratic polynomial. We have added a comment in the supplementary material (Figure 2) to make this clear.

We did not carry out cross-validation for three main reasons: 1) the data are too noisy to carry out cross-validation reliably; 2) cross-validation does not allow us to test the validity of specific model assumptions; 3) this study was not intended to compare different predictive models where cross-validation would provide more insights.  

If by “model selection” is meant “variables selection”, this has already been described in the manuscript. If by “model selection” is meant the selection of different spatial covariance models, this was not done because more complex spatial structures would require a larger amount of data. For example, we did not consider geostatistical model with directional effects, such as anisotropy, because of the large number of parameters that could not be estimated with precision from the available data.   

We used two sets of data for the current analysis, the fist was a mapping surveys in the western Ethiopia conducted between 2008-2010 and the other survey was conducted in 2013. The former was a secondary source and we don’t have the precise year for each location.  The distance between villages ranges from about 1 km to about 1,450 km. This has now been added to the main manuscript.

We have rephrased the sentence in question and replaced “the most parsimonious geostatistical model was fitted” with “A geostatistical model with an isotropic and exponential spatial covariance function was fitted”. We have already included a table with parameter estimates and 95% confidence intervals in the Supplementary Material. We have fitted a model without covariates which provides strongly similar predictions to the model with the aforementioned covariates. However, the model with covariates gives prevalence predictions with smaller standard errors. Hence, although S(x) mostly contributes to the point predictions of podoconiosis prevalence, the covariates still help to explain part of S(x) leading more accurate prevalence predictions.

We consider model validation as aimed at assessing the validity of specific modelling assumption in order to develop a credible probabilistic model for the data. Testing the validity of the adopted correlation function is fundamental in order to quantify uncertainty in prevalence estimates reliably (e.g. through the exceedance probabilities). Cross-validation would not help us to pursue this objective since this is intended to assess relative predictive performance which is outside the scope of the paper.

We have revised figure 2, 3 and 4 to show contrasting colours.  

**References**

Deribe K, Brooker SJ, Pullan RL, Sime H, Gebretsadik A, Assefa A, Kebede A, Hailu A, Rebollo MP, Shafi O, Bockarie MJ, Aseffa A , Reithinger R, Cano J, Enquselassie F, Newport MJ and Davey G. 2015a. "Epidemiology and Individual, Household and Geographical Risk Factors of Podoconiosis in Ethiopia: Results from the First Nationwide Mapping." Am J Trop Med Hyg 92(1):148–58.

Deribe K, Cano J, Newport MJ, Golding N, Pullan RL, Sime H, Gebretsadik A, Assefa A, Kebede A, Hailu A, Rebollo MP, Shafi O, Bockarie MJ, Aseffa A, Hay SI, Reithinger R, Enquselassie F, Davey G and Brooker SJ. 2015b. "Mapping and Modelling the Geographical Distribution and Environmental Limits of Podoconiosis in Ethiopia." PLoS Negl Trop Dis 9(7):e0003946.

Farr TG and Kobrick M. 2000. "Shuttle Radar Topography Mission Produces a Wealth of Data." Amer. Geophys. Union Eos 81(48):583-85.

Hengl T, Mendes de Jesus J, Heuvelink GBM, Ruiperez Gonzalez M, Kilibarda M and et al. 2017. "Soilgrids250m: Global Gridded Soil Information Based on Machine Learning." PLoS One 12(2):e0169748.

Price E. 1990. "Podoconiosis:Non-Filarial Elephantiasis." Oxford Medical Publications, Oxford, UK.
